# Erector spinae plane block as an alternative to caudal block in concurrent pediatric urologic and inguinal surgery: A double-blinded randomized controlled trial

**DOI:** 10.1097/MD.0000000000042109

**Published:** 2025-04-11

**Authors:** Volkan Özen, Engin İhsan Turan, Taylan Kirdan, Tahir Arda Ayas, Ela Erten, Serap Karacalar

**Affiliations:** a Department of Anesthesiology and Reanimation, Prof. Dr. Cemil Tascioglu City Hospital, Istanbul, Turkey; b Department of Anesthesiology, Istanbul Health Science University, Kanuni Sultan Süleyman Education and Training Hospital, Istanbul, Turkey; c Department of Anesthesiology and Reanimation, Gülhane Research and Training Hospital, Istabul, Turkey.

**Keywords:** caudal block, erector spinae block, postoperative analgesia, ultrasound

## Abstract

**Background:**

The objective of this study is to evaluate and compare the analgesic effect of ultrasound (US)-guided a high-volume bilateral erector spinae plane (ESP) block with that of US-guided caudal block (CB) in these surgeries.

**Methods:**

This prospective, randomized, double-blind study was conducted with 60 male patients, aged 1 to 7 years, who underwent lower abdominal surgery and circumcision concurrently. The patients were randomized into 2 groups: ESP and CB. US-guided ESP block at the L4 vertebral level was performed preoperatively using 1 mL/kg 0.125% bupivacaine (maximum of 20 mL), which was applied to patients in the ESP group. US-guided CB was performed preoperatively using 1 mL/kg 0.125% bupivacaine (max 20 mL), which was applied to patients in the CB group. Face, legs, activity, cry, and consolability scores for pain were recorded at 30 minutes and 1, 2, 4, 6, 12, and 24 hours postoperatively. Analgesic requirements, time to first analgesic requirement, and postoperative complications were also documented.

**Results:**

The 6- and 12-hours postoperative face, legs, activity, cry, and consolability scores were lower in the ESP group (*P* = .011, *P* = .021, respectively). The number of analgesic requirements in the first 24 hours postoperatively was significantly lower in the ESP group (*P* = .002). No postoperative complications were observed in either of the groups.

**Conclusion:**

This study shows that the ESP block provides effective and safe postoperative analgesia compared to the CB in pediatric patients undergoing circumcision and lower abdominal surgeries. Clinicians may consider the ESP block as another option for CB in such surgeries based on their clinical experience.

## 1. Introduction

Families of male children who are about to undergo lower abdominal surgery, such as inguinal herniorrhaphy or orchiopexy, prefer circumcision, which is commonly performed for cultural and religious reasons in our country, to be performed in the same session while the child is under general anesthesia. Circumcision and lower abdominal surgery in the pediatric population result in a painful postoperative period even when each procedure is evaluated separately.^[1,2]^

Caudal block (CB), a regional anesthesia technique, is the most commonly used neuraxial block method for postoperative pain control in subumbilical surgeries in children.^[3,4]^ However, peripheral nerve blocks have recently become preferred, because they provide longer and safer analgesia.^[5]^ One of these blocks, the erector spinae plane (ESP) block, has been shown to provide effective postoperative analgesia when administered from the lumbar level for sacral, lower abdominal, and urogenital surgeries in pediatric patients.^[6]^ When the ESP block is administered from the lower lumbar level with a high volume in adult patients, the local anesthetic (LA) also spreads to the sacral area through the craniocaudal direction.^[7]^ Based on this information, the ESP block can be used as an alternative to CB to provide postoperative analgesia in urological and inguinal surgeries performed during the same session in male children.

In this double-blind, randomized controlled study, we hypothesized that the ultrasound (US)-guided ESP block would provide more effective and longer postoperative analgesia than CB in pediatric patients undergoing unilateral lower abdominal surgery together with circumcision. The primary aim of this study was to compare the analgesic efficacy of ESP and CB in pediatric circumcision and lower abdominal surgeries, based on postoperative pain scores. The secondary endpoints of this study were the time to first analgesic requirement and postoperative complications.

## 2. Methods

### 2.1. Study design and participants

This double-blind randomized controlled trial was performed in accordance with the principles of the Declaration of Helsinki at the Anesthesiology and Reanimation Service of an Education and Research Hospital between February and November 2021. This study was approved by the Clinical Ethics Committee (Decision No: 2021.42; Decision Date: January 4, 2021) and registered at clinicaltrials.gov (NCT05284734). After obtaining written consent from the parents of the participants, patients aged 1 to 7 years belonging to the American Society of Anesthesiologists I–II group, who were scheduled to undergo circumcision and unilateral lower abdominal surgery in the same session, were included in the study. Lower abdominal surgery was classified as inguinal hernia repair, orchiopexy, or hydrocelectomy.^[2]^ Patients aged <1 year and >7 years; patients with a neurological deficit, bleeding diathesis, or a history of allergy to LA drugs; patients with redness or infection on physical examination in the area where the injection was administered; and patients with any congenital lumbar anomaly, mental retardation, psychiatric disorder, or liver and/or kidney disorder, in addition to those who refused to participate, were excluded from the study. Although these surgical procedures are usually performed as day surgeries when performed separately in our hospital, surgical procedures in 2 different regions are followed up in the hospital for the first 24 hours postoperatively in accordance with the hospital policy.

### 2.2. Sample size estimation

**Figure 1.**
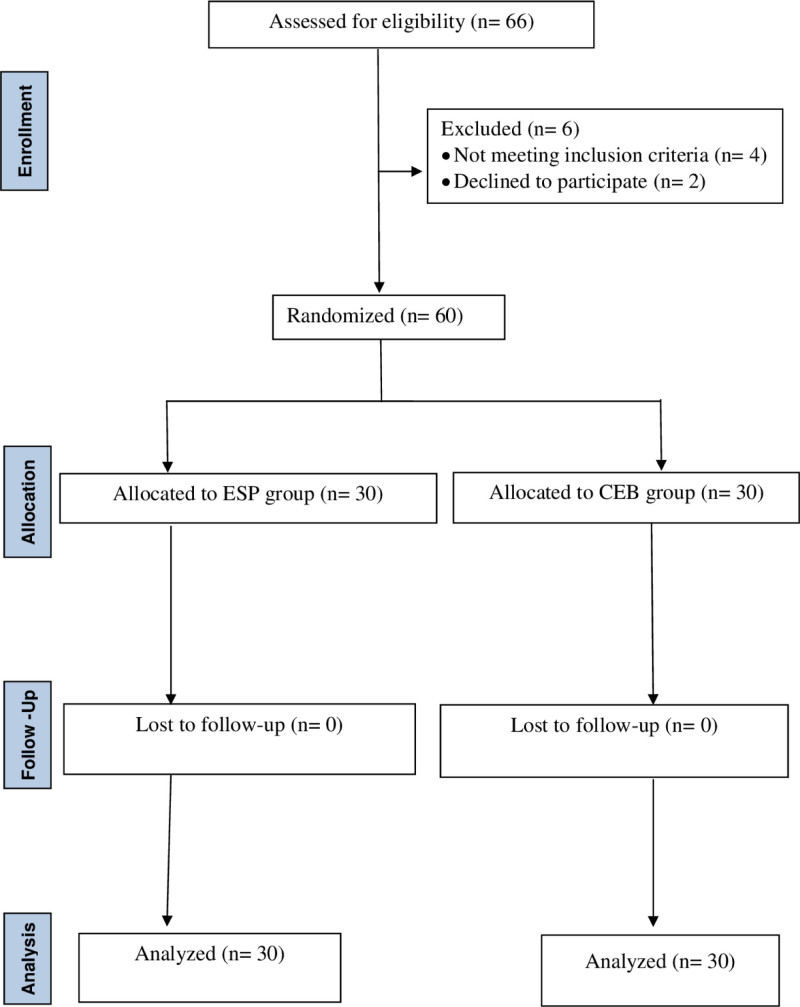
Flow of participants through trial.

The primary outcome, which was the number of patients who required analgesics over a 24-hours period, was assessed using a sample size generated by G* Power 3 based on a pilot study conducted among 5 patients each in the ESP and CB groups. The proportion of patients who required analgesics were 0.2 and 0.60 in the ESP and CB groups, respectively. The sample size was calculated at a power of 95% and significance level of 5%. Finally, 30 patients in each group and 60 patients in both groups were required to obtain statistically significant values. Considering the possibility of patient dropout due to various reasons during the study, 66 participants (33 in each group) were included. The 2010 CONSORT statement for randomized trials of nonpharmacological treatments was used as a guide to report the results (Fig. 1).

### 2.3. Randomization and masking

Randomization was performed using a computer-generated randomization table (http://www.random.org), and patients were divided into 2 groups: ESP and CB. The nurse was blinded to the face, legs, activity, crying, and consolability (FLACC) scores. An anesthesiologist was blinded to the data collection regarding analgesic requirement and postoperative complications. Transparent sterile drapes were applied to the injection sites, and both blocks were administered to all patients to evaluate postoperative complications. Thus, the anesthetist was blinded to the postoperative complications.

### 2.4. General anesthesia procedure

All patients were administered 0.5 mg/kg of oral midazolam as premedication. Standard monitoring was performed in the operating room. The patient was administered intravenous (IV) propofol (2 mg/kg) and fentanyl (0.5 µg/kg) for general anesthesia induction. After loss of eyelash reflex, a laryngeal mask suitable for the weight and age of the patient was attached to the anesthesia device without neuromuscular blockade. Anesthesia was maintained using 2% sevoflurane in a mixture of 50% air and oxygen. Demographic information such as age, weight, type of surgery, duration of the procedure, and complications were recorded.

### 2.5. Regional anesthesia procedure

All blocks were performed with the patient in the left lateral decubitus position following the induction of general anesthesia and airway control.

### 2.6. CB procedure

**Figure 2.**
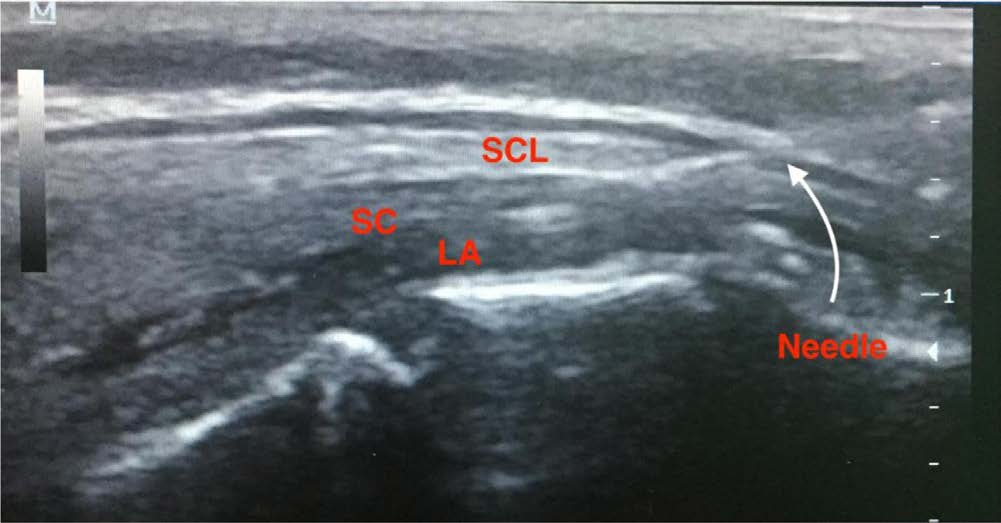
Caudal block.

Povidone–iodine was used to sterilize the surgical area. The linear US probe was covered with sterile plastic cover and gel. The sacral hiatus was visualized at 5 to 10 MHz using an out-of-plane transverse view at the sacral cornus level. Next, the linear probe was placed longitudinally at the midline following a 90° rotation to evaluate the sacral cornus, sacrococcygeal ligament, and sacral bone. A 22-G 50-mm echogenic block needle was placed longitudinally through the sacrococcygeal membrane into the sacral canal using the in-plane technique (Fig. 2). Lack of blood or cerebrospinal fluid on aspiration was confirmed, and 0.125% bupivacaine was administered at a dose of 1 mL/kg (limited to a maximum dose of 20 mL per side). During the LA injection, Doppler US was used to observe any dilation of the caudal epidural space or turbulent flow.

### 2.7. ESPB procedure

**Figure 3.**
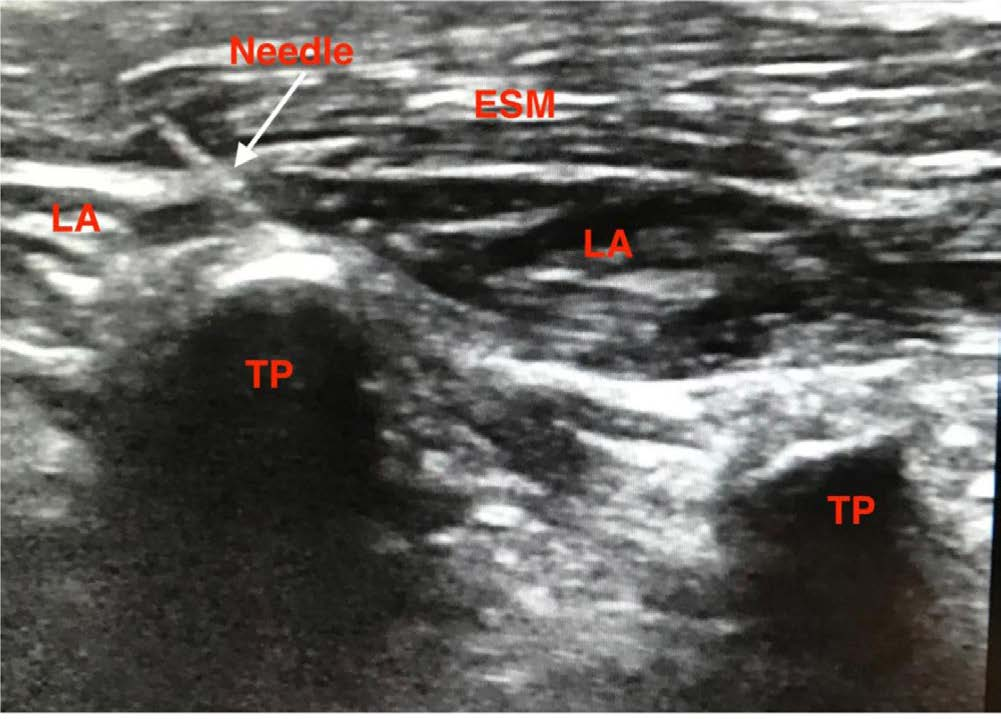
Erector spine plane block.

An ESP block was performed following the induction of general anesthesia at the level of the L4 transverse process. Bilateral antiseptic preparation of the block sites was followed by the application of a linear probe (5–10 MHz) 2 to 3 cm lateral to the spine at L4. The erector spinae muscle and transverse process were identified, and a 22-G, 80-mm echogenic block needle was advanced toward the transverse process until contact was established. Following hydrodissection, 1 mL/kg 0.125% bupivacaine (limited to a maximum dose of 20 mL per side) was injected deep into the erector spinae (Fig. 3). LA spread in both the cephalad and caudal directions was observed. The same procedure was repeated for the contralateral side.

Block administration was followed by placing the patient in the supine position. Surgery was initiated 15 minutes later.^[8]^ The heart rate and noninvasive arterial blood pressure measurements were recorded every 5 minutes before and after block administration. If the heart rate and blood pressure increased by 20% or more after the surgical incision, the block was considered unsuccessful, and 0.5 μg/kg of fentanyl IV administration at a planned. Complications that occurred during the procedure were recorded.^[1]^

Following the completion of the surgery, the patient was transferred to the recovery room, where the heart rate, blood pressure, peripheral oxygen saturation value, pain score, and side effects were recorded.

### 2.8. Postoperative follow-up

#### 2.8.1. Measures

*FLACC score:* FLACC scores were recorded 30 minutes postoperatively and at 1, 2, 4, 6, 12, and 24 hours post-surgery. Rescue analgesia was planned based on the FLACC score. Paracetamol IV at a dose of 10 mg/kg was administered if the score was between 2 and 4, and tramadol IV at a dose of 1 mg/kg was administered if the score was >4.^[2]^ The analgesic dose and time of administration were also recorded. The FLACC scale was used by nurses (who were blinded to the study) to evaluate pain in both the postoperative recovery room and the ward. The scale has been used routinely for the last 3 years by the recovery unit and ward nurses for postoperative pain evaluation following pediatric surgery.

#### 2.8.2. Primary outcome

*Analgesic requirements:* Analgesic requirements in the first 24 hours postoperatively and time to the first analgesic were recorded at the postoperative follow-up visits (30 minutes and 1, 2, 4, 6, 12, and 24 hours) by an anesthesiologist blinded to the study groups.

#### 2.8.3. Secondary outcomes

*Analgesic efficacy:* Analgesic efficacy was evaluated based on postoperative pain scores using the FLACC score.

*Postoperative complications:* The presence of urinary retention, motor block, and ecchymosis or hematoma at the injection site was evaluated during the postoperative period (30 minutes and 1, 2, 4, 6, 12, and 24 hours) by an anesthesiologist who was blinded to the study groups.

The modified Bromage scale (0, no motor block; 1, able to move the legs; and 2, unable to move the legs) was used to evaluate lower-extremity motor block.^[9]^

Urinary retention was defined as a distended palpable bladder associated with pain.^[9,10]^

Ecchymosis is defined as discoloration of the skin caused by the infiltration of blood into the subcutaneous tissues or breakage of the subcutaneous capillary vessels.

Hematoma was defined as abnormal swelling or hardening caused by the accumulation of blood (a hematoma occurs when there is hardening, regardless of whether there is discoloration of the skin).^[11]^

### 2.9. Statistical analysis

The data were analyzed using IBM SPSS Statistics for Windows (version 22.0; IBM Corp., Armonk, NY). The compliance of the values with a normal distribution was evaluated using the Shapiro–Wilk test. Descriptive statistics for continuous variables are presented as mean ± standard deviation and median with interquartile range (median). Categorical variables were presented as absolute numbers and frequencies. In exploratory data analysis, differences between groups were determined using a two-sample *t* test or nonparametric rank-based Mann–Whitney *U* test for skewed data, as well as differences in mean or median with 95% confidence intervals (CI) (difference in mean/median [95% CIs]). We calculated 95% CIs for the difference in median using bootstrapping and 95% CIs for the difference in frequencies as approximate CIs for the risk difference. To test whether the frequency of complications differed between the 2 groups, *χ*^2^-tests or Fisher exact tests were performed. OR with 95% CIs (OR [95% CIs]) were provided. The effect size was calculated using the Cohen method. Statistical significance was set at *P* < .05.

## 3. Results

Of the 66 patients initially assessed for eligibility, 60 were included in the final analysis (Fig. 1). There were no statistically significant differences between the groups based on the descriptive features of the patients, such as age, weight, operation duration, operation type, or the presence of urinary retention. Urinary retention was observed in 6 patients in the CB group (Table 1).

**Table 1:** Descriptive features of the patients.

Variables	Caudal block group (n = 30)	Erector spina plane block group (n = 30)	Difference 95% CI
Age (yr)	4 [3–5]	4 [3.75–5.25]	3 [2.0–3.5]
Weight (kg)	16.50 [12.75–19.25]	16 [12.75–20.25]	12 [9.5–14.0]
Operation duration (min)	38 [34–41.25]	39.50 [38–41]	27 [20.50–34.0]
Operation type			–
Circumcision + inguinal hernia (unilateral)	12 (40.0)	11 (36.7)	
Circumcision + hydrocelectomy (unilateral)	4 (13.3)	3 (10.0)	
Circumcision + orchiopexy (unilateral/bilateral)	14 (46.7)	16 (53.3)	
Circumcision + orchiopexy			
Unilateral	11 (78.6)	12 (75)	1.0 [0.70–1.55]
Bilateral	3 (21.4)	4 (25)	0.8 [0.2–3.1]
Presence of urinary retention (yes/no)	6/24	0/30	-

Data are expressed as median [percentiles 25–75] or n (%).

CI = confidence interval.

The 6- and 12-hours postoperative FLACC scores were lower in the ESP group (*P* = .011, *P* = .021, respectively), while the 30 minutes and 1-, 2-, 4-, and 24 hours FLACC scores were not significantly different between the groups (*P* > .05) (Table 2).

**Table 2:** Postoperative FLACC scores at different time intervals.

FLACC	Caudal block group	Erector spina plane block group	*P*-value	Effect size
2 h	0 (0–0)	0 (0–0)	.078	0.227
4 h	0 (0–0)	0 (0–0)	.078	0.227
6 h	0 (0–3)	0 (0–0)	**.011**	0.330
12 h	0 (0–2)	0 (0–0)	**.021**	0.298
24 h	0 (0–0)	0 (0–0)	.078	0.227

Data are reported as median (IQR), and effect size values were calculated with the Cohen method.

FLACC = face, legs, activity, cry, and consolability.

The time to first analgesic requirement in the first 24 hours postoperatively was significantly lower in the CB group (*P* = .022). Total analgesic consumption was significantly lower in the ESP group (*P* = .043). Twelve patients in the CB group used analgesics (acetaminophen); 2 of them used analgesics twice, and 10 patients used them once. Only 2 patients in the ESP group used analgesics (acetaminophen), and both patients used them only once. There was a statistically significant difference in analgesic use between CB group and ESP group (12 [0%] vs 2 [6.7%]; *P* = .002; OR = 9.33 [1.4; 24.54]) (Table 3). None of the patients required fentanyl or tramadol administration.

**Table 3:** Postoperative analgesic requirements.

Variables	Caudal block group (n = 30)	Erector spina plane block group (n = 30)	Difference 95% CI	*P*-value
Analgesic use (yes/no)				.002
Yes	12 (40)	2 (6.7)	6 [1.4–24.54]	
No	18 (60)	28 (93.3)	0.6 [0.4–0.8]	
Time to first analgesia (h)	6 [4–6]	22.65 [22.30–23]	6 [4.0–11.5]	.022
Total analgesic consumption (mg)	205 [180–275]	125 [90–160]	190 [160–280]	.043

Data are expressed as median [percentiles 25–75] or number.

CI = confidence interval.

No complications such as nausea, vomiting, bradycardia, hypotension, or allergic reactions were observed during the perioperative period in either group.

## 4. Discussion

To the best of our knowledge, this is the first randomized controlled prospective study comparing ESP block and CB for postoperative pain relief after circumcision and lower abdominal surgery in children. The results of our study demonstrated that the ESP block provided more effective pain relief and prolonged analgesia than CB and did not result in any complications.

Truncal peripheral nerve blocks are commonly preferred because of their long-term analgesic effects and low incidence of complications.^[12–14]^ Although we did not find any studies comparing CB and ESP blocks in the literature, few studies have compared other US-guided interfascial nerve blocks with CB in the pediatric population. A previous study on various urological surgeries, in which a lateral quadratus lumborum (QL) block was compared with a CB, found no difference between the groups in terms of pain scores at 1, 4, and 24 hours postoperatively, although LA was used at the same concentration and dose in both blocks.^[15]^ Another study comparing the transversus abdominis plane block and CB found no difference between the postoperative pain scores in the interfascial block group despite the use of a lower LA volume.^[16]^ The pain score was found to be lower in the posterior QL group in another study where posterior QL block and CB were compared (0.7 mL/kg of 0.25% bupivacaine was administered in both groups).^[13]^ Our study found that the FLACC pain scores were lower in the ESP group at 6 and 12 hours postoperatively. The lack of a significant difference between the groups in the initial 4-hours pain scores in the current study could be attributed to the effectiveness of both block types in the postoperative period, while the lack of a significant difference in the 24-hours pain scores could be due to the administration of a larger volume of additional analgesic drugs in the CB group than in the ESP group.

Evidence regarding the relationship between ESP block volume and dermatomal spread in pediatric patients is inadequate and inconclusive. Although a systematic review analysis emphasized that a volume corresponding to a dose of 0.1 mL/kg should be administered for each dermatome area in pediatric ESP blocks, a LA volume of 0.2 to 1 mL/kg unilaterally and 0.3 to 0.5 mL/kg bilaterally was used, despite variance according to the level used.^[17]^ In contrast to our study, Aksu et al^[2]^ compared QL and ESP blocks using a high concentration and low volume of LA, whereas El-Imam et al^[8]^ compared an ilioinguinal/iliohypogastric nerve block and an ESP block using LA at the same concentration but low volume in the 2 groups, similar to our study. The duration of analgesic efficiency was longer with ESP block in both studies. The first additional analgesic drug requirement occurred earlier in the postoperative period in the CB group, despite the use of a higher volume of LA, in a study where CB and ilioinguinal/iliohypogastric nerve blocks were compared for inguinal region surgery in pediatric patients.^[18]^ The total amount of additional analgesic drug used in the current study was lower in the ESP group than in the CB group due to the longer-lasting and more effective postoperative analgesia provided. However, the mechanisms underlying the observed clinical effects of the ESP block remain controversial, and further investigations into the precise mechanisms of the block are required.^[19,20]^ It is hypothesized that the multi-dermatomal sensory block experienced is due to cranial and caudal spread of the injected LA during the ESP block. Govender et al^[21]^ stated that the LA administered in their study was believed to spread in the craniocaudal direction and to the paravertebral, epidural, and intercostal areas via the intertransverse connective tissue. In a study by Celik et al^[22]^ high-dose LA administered from the L4 level spread to the epidural area and stimulated T10–S2 dermatomes, which were observed during magnetic resonance imaging examination. However, Alici et al^[7]^ reported that the lumbar and sacral plexuses were blocked by a lumbar ESP block with a higher dose volume than that of the L3 level. Therefore, we believe that the high-dose LA administered in this study and ESP applied at the lumbar level in pediatric patients spread to the epidural area through the connective tissue and blocked the branches of the lumbar and sacral spinal nerves. Although rarely encountered, CB has been reported to cause block-related complications such as hypotension, cardiac arrest, seizure, and motor block, which may have serious consequences, and needle-related complications such as bone puncture, sacral pain, intravascular injection, dural puncture, and total spinal anesthesia.^[1,13]^ While urinary retention was observed in 3 patients in the CB group in the study by Ipek et al^[23]^ no CB-related complications were observed in the study by Oksuz et al.^[13]^ The reason for the urinary retention observed in 6 patients in the CB group in this study could be because the LA was administered at a higher volume than in other studies.^[13,23]^ Moreover, US was reported to be ineffective in decreasing the complication rate in an observational study of 18,950 patients from the database of the Pediatric Regional Anesthesia Network, while recent randomized controlled studies have found that US decreases the incidence of needle-related complications.^[24–27]^ No needle-related complications. This could be due to the ability to distinguish the anatomical structures of the sacral region using US and the penetration of the sacrococcygeal ligament while observing the entire needle in real-time using the in-plane technique during CB administration. A few complications of the ESP block have been reported in the literature. In a study by Tulgar et al, quadriceps muscle weakness, which continued for 14 hours postoperatively following the ESP block, was reported in a patient in whom the block was administered at the T11 level.^[28]^ A possible because of the spread of the block to the lumbar plexus due to the administration of more than 1 LA at high concentrations and volumes. The use of the in-plane technique while performing an US-guided ESP block could therefore prevent complications, as the entire needle can be observed while being advanced into the transverse process in real-time.^[6,29]^ We believe that the reason for the absence of ESP block-related complications in the current study was the administration of ESP using the in-plane technique and not the LA at high concentrations and volumes.

LA systemic toxicity (LAST) remains an important complication in pediatric patients, although it occurs in a limited number of cases on a case-by-case basis. As regional anesthesia techniques are administered under general anesthesia in children, it is difficult to detect signs of LA-induced LAST, such as convulsions. Therefore, monitoring the symptoms of rhythm disturbances and cardiovascular collapse is necessary in pediatric patients undergoing regional anesthesia. In addition, the appropriate choice of agent, aspiration before each injection, and controlled administration of LAs using US reduce the risk of developing LAST.^[30]^ None of the patients who underwent blocks had LAST-related symptoms (rhythm disturbances, such as ventricular tachycardia, ventricular fibrillation, or cardiovascular collapse). This may be attributed to the use of 0.125% bupivacaine at 1 mL/kg and an upper limit of 20 mL when performing the blocks in our patients, especially in bilateral ESP blocks. The dose used was of low concentration, and the upper limit was set according to the maximum recommended dose of bupivacaine (2.5 mg/kg), which is not exceeded according to current guidelines.^[31]^

### 4.1. Limitations

Our study had some limitations. First, it is possible that the findings are unique to our practice and not necessarily generalizable to other centers. Second, since the CB and ESP blocks were administered to pediatric patients after general anesthesia in the current study, sensory dermatomal level evaluations of both blocks were not performed preoperatively. Third, the results of this study are applicable to children aged 1 to 7 years who have undergone abdominal surgery and circumcision, and may not be generalizable to patients of other age groups.

## 5. Conclusions

This study demonstrated that the ESP block can provide significantly more effective and longer-lasting analgesia than CB in the management of multimodal analgesia in children undergoing circumcision and lower abdominal surgery in the same session. We believe that ESP block is safer than CB, with an expected lower risk of complications. Therefore, we suggest that clinicians use the ESP block as an alternative to CB, depending on their clinical knowledge and experience.

## Author contributions

**Data curation:** Engin İhsan Turan, Tahir Arda Ayas, Ela Erten.

**Formal analysis:** Tahir Arda Ayas, Ela Erten.

**Methodology:** Volkan Özen, Serap Karacalar.

**Project administration:** Volkan Özen.

**Supervision:** Volkan Özen, Engin İhsan Turan, Taylan Kirdan.

**Visualization:** Tahir Arda Ayas, Ela Erten.

**Writing – original draft:** Volkan Özen, Tahir Arda Ayas, Serap Karacalar.

**Writing – review & editing:** Volkan Özen, Engin İhsan Turan, Taylan Kirdan.
